# Influence of Nanopore Shapes on Thermal Conductivity of Two-Dimensional Nanoporous Material

**DOI:** 10.1186/s11671-016-1649-5

**Published:** 2016-09-26

**Authors:** Cong-Liang Huang, Zun Huang, Zi-Zhen Lin, Yan-Hui Feng, Xin-Xin Zhang, Ge Wang

**Affiliations:** 1 School of Electric Power Engineering, China University of Mining and Technology, Xuzhou, 221116 China; 2 School of Mechanical Engineering, University of Science and Technology Beijing, Beijing, 100083 China; 3 School of Materials Science and Engineering, University of Science and Technology Beijing, Beijing, 100083 China

**Keywords:** Thermal conductivity, Nanoporous material, Two-dimensional material, Electron mean free path

## Abstract

The influence of nanopore shapes on the electronic thermal conductivity (ETC) was studied in this paper. It turns out that with same porosity, the ETC will be quite different for different nanopore shapes, caused by the different channel width for different nanopore shapes. With same channel width, the influence of different nanopore shapes can be approximately omitted if the nanopore is small enough (smaller than 0.5 times EMFP in this paper). The ETC anisotropy was discovered for triangle nanopores at a large porosity with a large nanopore size, while there is a similar ETC for small pore size. It confirmed that the structure difference for small pore size may not be seen by electrons in their moving.

## Background

**Fig. 1:**
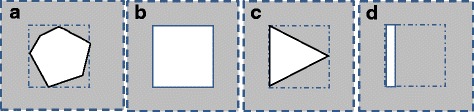
Different nanopore shapes. **a** Ordinary nanopore. **b** Square nanopore. **c** Triangle nanopore. **d** Slit nanopore

The lattice thermal conductivity (LTC) of a nanoporous material has already been widely studied [1–7], with Boltzmann transport equation, molecular dynamics simulation, Monte Carlo simulation, and some other methods. But the electronic thermal conductivity (ETC) was scarcely studied yet [8, 9]. The ETC of metallic nanoporous materials (MNM) under the influence of nanopore shapes was studied in this paper. If phonons are treated as particles in a free-gas model category and the phonon dispersion relations were not fully taken into account, there will be an approximately similar decreasing tendency of the LTC versus porosity with that of the ETC for metallic nanoporous materials. Thus, the result of the ETC got in this paper can be approximately extended to the total thermal conductivity. The shape of nanopores in a nanoporous material is usually irregular, like that in Fig. 1a, not regular like that in Fig. 1b or c or d. It is time-consuming to simulate a nanoporous material with irregular shape nanopores (each nanopore may possess a different nanopore shape). Some typical nanopore shapes, shown as in Fig. 1b–d, were selected to probe their influence on the ETC. We expect that the result for typical nanopore may give some important information about the influence of nanopore shape. A similar nanopore distribution was applied in this paper to get rid of the distribution influence. And a simulation method developed in our previous work [9–12] was applied here to predict the ETC. More about the simulation method was summarized in Methods. The simulation result was discussed in Results and Discussion.

## Methods

While the specific heat contributed by electrons in an MNM is equal to that in the bulk material [13], following kinetic theory, the reduced ETC will equal to the reduced electron mean free path (EMFP) [14], i.e., *k*_e_* = *l*_e_*, where *k*_e_ is the ETC, *l*_e_ is the EMFP, the superscript “*” in this paper indicates a dimensionless quantity scaled by that of the corresponding bulk quantity. While a linear relationship (*k*_e_* = *l*_e_*) exists between the ETC and the EMFP, only the EMFP should be obtained. A simulation method based on the kinetic theory has already been set up to predict the EMFP in our previous work [9–12]. This simulation method was also applied in this work to predict the EMFP. For consistency, the hypotheses applied in the simulation method were summarized here: (a) The free-electron-gas model (also denoted as the Drude model) [15] was applied. (b) Each electron moves along a straight line at the Fermi velocity until terminated at a boundary surface or after a sufficiently long path has been traveled [16, 17]. (c) Only the *Z* component of free path contributes to the EMFP. Based on the similar hypotheses, an EMFP calculation model was also set up for a hollow nanowire in Ref. [18].

**Fig. 2:**
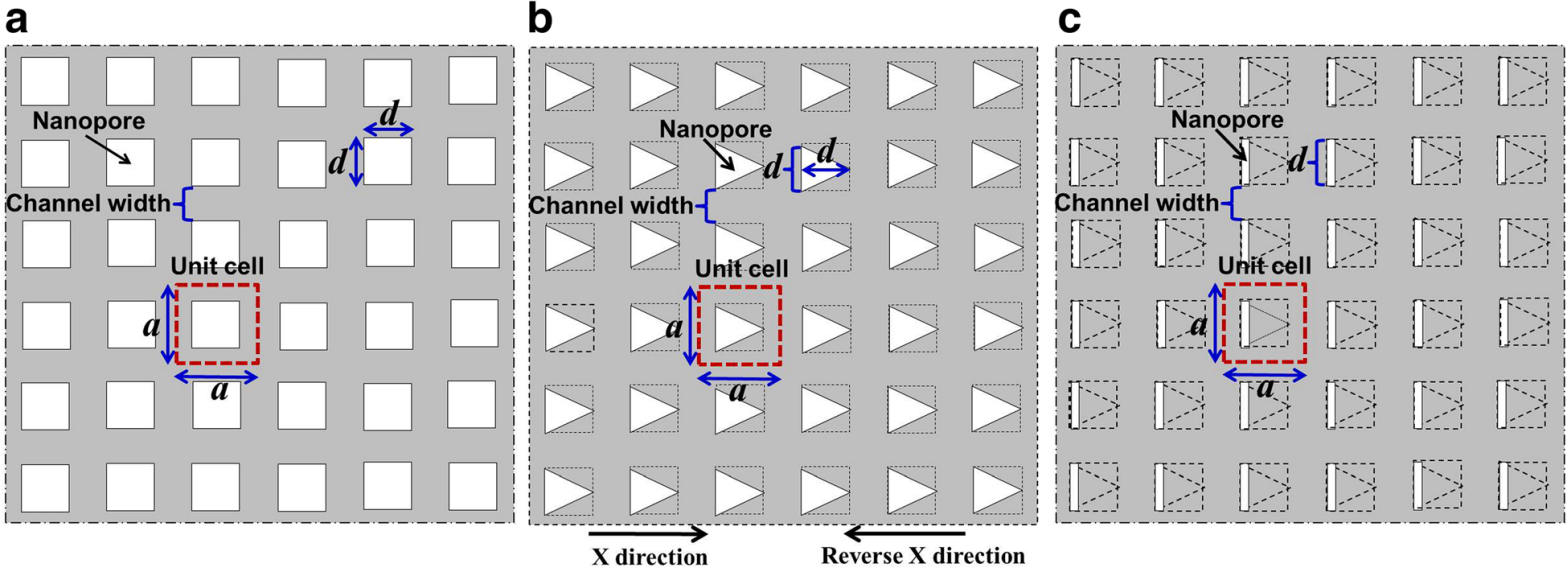
MNMs with different nanopore shapes. **a** Square nanopore. **b** Triangle nanopore. **c** Slit nanopore. Here, *d* is the side length of a square nanopore and *a* is the distance between the centers of two adjacent pores

With the simulation method applied, the ETCs of MNMs with triangle nanopores and slit nanopores were, respectively, simulated for comparison with that of MNMs with square nanopores. Inelastic boundary conditions were applied in the simulation. The ETC for square nanopore was referred from our previous work [9]. For comparison, a similar nanopore distribution and a similar channel width (same *a* and *d* in Fig. 2) were applied for different nanopore shapes. Slit pore in Fig. 2c was set to be very thin with width equal to one tenth of length *d*. Porosities were calculated by *φ* = *d*^2^/(*a*^2^) for square nanopore, *φ* = *d*^2^/(2*a*^2^) for triangle nanopore, and *φ* = *d*^2^/(10*a*^2^) for slit nanopore, where *d* is the side length of a square nanopore and *a* is the distance between the centers of two adjacent pores. The largest porosity of MNMs with slit nanopores and triangle nanopores will be no larger than 10 and 50 %, respectively.

## Results and Discussion

**Fig. 3:**
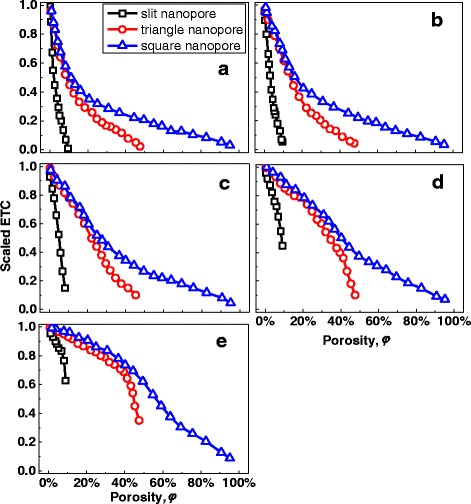
ETC with different porosity. **a** With d* = 1/4. **b** With d* = 1/2. **c** With d* = 1. **d** With d* = 2. **e** With d* = 4

**Fig. 4:**
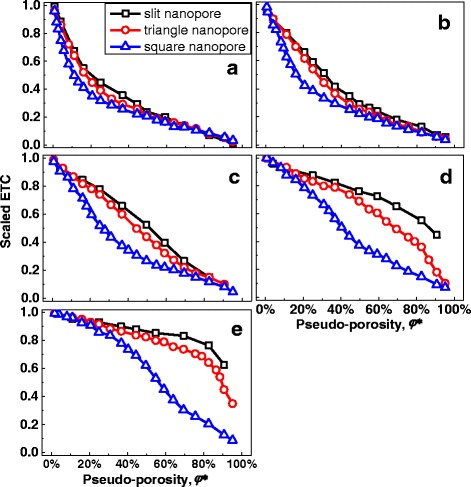
ETC with different pseudo-porosity. **a** With d* = 1/4. **b** With d* = 1/2. **c** With d* = 1. **d** With d* = 2. **e** With d* = 4

The ETC for different nanopore shapes was shown in Fig. 3. It shows that the ETCs for MNMs with slit nanopores and triangle nanopores are much smaller than that for MNMs with square nanopores at the same porosity. At porosity *φ* ≈ 10 %, the scaled ETC of MNMs with triangle nanopores or square nanopores will be larger than 0.5, while that of MNMs with slit nanopores is smaller than 0.1. Figure 3 tells that with same porosity, the ETC will be quite different for different nanopore shapes. This result is obvious, because the thermal transport channel width will be quite different for three different nanopore shapes at the same porosity. And the channel width will greatly affect the ETC. To eliminate the influence of channel width, a pseudo-porosity was defined as φ* = *d*^2^/*a*^2^ for all three different nanopore shapes to make sure the comparison was made under the same channel width. Obviously, same pseudo-porosity means same channel width, and there is a same pseudo-porosity for three different nanopore shapes in Fig. 2 for example. The pseudo-porosity is also the true porosity for square nanopores. The ETC versus pseudo-porosity was shown in Fig. 4. From Fig. 4, it can be drawn that with same channel width, the nanopore shape has little influence on ETC at small pore size, because the scattering caused by a small nanopore may like a defect-scattering while the structure difference between different nanopore shapes for small nanopores is difficult to be seen by electrons in their moving. For large pore size, the ETC for different nanopore shapes will be quite different. This can be easily understood by that the nanopore shape becomes large to be seen by electrons in their moving. It can be concluded that with same channel width, the influence of different nanopore shapes can be approximately omitted if the nanopore is small enough (smaller than 0.5 times EMFP in this paper).

**Fig. 5:**
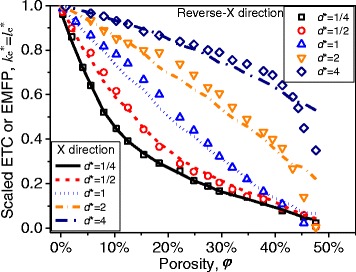
ETC of MNMs with triangle nanopores along *X* and reverse-*X* directions

**Fig. 6:**
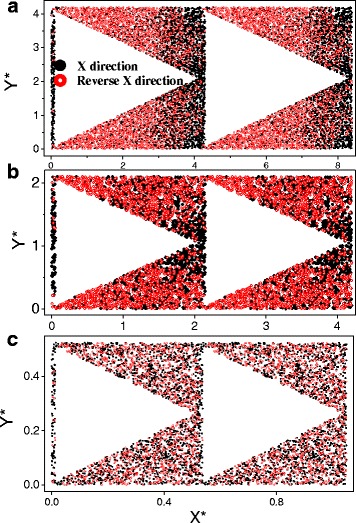
Electron distributions in MNM with *φ* = 45.4 %. **a** d* = 4. **b** d* = 2. **c** d* = 0.5. X* and Y* signify the coordinate axis, with values scaled by the bulk EMFP. The *solid black circle* and the *hollow red circle* represent the electron locations along *X* and reverse-*X* directions, respectively

ETCs of MNM with triangle nanopores along *X* and reverse-*X* directions were compared in this part. The data are expressed according to the scaled pore size d* = *d*/*l*_0_ and the scaled ETC (reduced by the bulk ETC); *l*_0_ is the EMFP of the bulk material. The result was shown in Fig. 5. For most pore size *d*, there is not a linear relationship between ETC and the porosity, different from that happens in the microscale. This phenomenon was also discovered in our previous work [9]. As is to be expected, a larger nanopore size will lead to a larger ETC while porosity is fixed [9]. This is quite similar with the result got for LTC [1, 19, 20]. It shows that there is not an obvious ETC difference along *X* and reverse-*X* directions except at high porosity for large nanopore size. It can be understood by that the similar channel width should be responsible for the equal ETC along *X* and reverse-*X* directions at low porosity, while the nanopore can be treated as only defects. But for the MNMs with large porosity and large nanopore size, the structure difference between *X* and reverse-*X* directions can be seen by electrons in their moving while the size is large enough, so there will be a different ETC at large porosity in Fig. 5. This validates the result on the last paragraph that a shape difference for large nanopores will be seen by electrons in their moving. To further illustrate the different scattering effects for *X* and reverse-*X* directions, the electron distributions were shown in Fig. 6. The solid black circle and the hollow red circle in Fig. 6 represent the electron locations along *X* and reverse-*X* directions at a given time, respectively. The large electron distribution difference between *X* and reverse-*X* directions in Fig. 6a, b confirmed that there will be different electron scatterings for the MNM with a large porosity and a large pore size. And electrons along reverse-*X* directions distribute more uniformly than that along *X* direction in Fig. 6. It means that the electron transfer along reverse-*X* direction will be easier than along *X* direction. Thus, there is a larger ETC for reverse-*X* direction than for *X* direction in Fig. 5. For the MNM with a large porosity but a small pore size, the electron distribution looks similar along *X* and reverse-*X* directions in Fig. 6c. It confirmed that the structure difference for small pore size d* = 0.5 is too small to be seen by electrons in their moving.

## Conclusions

In this paper, the influence of nanopore shapes on the ETC of two-dimensional MNMs was studied with a method setup in our previous work. Three typical shapes were selected to probe their influence. While the ETC can be also affected by the nanopore distributions, a similar nanopore distribution was applied to get rid of their influence. The result shows that different nanopore shape will lead to different ETC at same porosity. This can be easily understood by that different nanopore shape with same porosity will lead to a different electron transfer channel width. The different channel width should be responsible for the difference. So a further study was carried out with a same channel width. Results tell that the nanopore shape has little effect on ETC at small pore size, because the scattering caused by a small size nanopore may like a defect-scattering while the structure difference between different nanopore shapes with small pore size is difficult to be seen by electrons in their moving. For large pore size, the ETC for different nanopore shapes will be quite different at a large porosity. This can be easily understood by that the nanopore shape becomes large to be seen by electrons in their moving. It can be concluded that the nanopore shapes can be approximately omitted if the nanopore is small enough (smaller than 0.5 times EMFP in this paper). The ETC anisotropy was discovered for triangle nanopores at a large porosity with a large nanopore size, while there is a similar ETC for small pore size. It confirmed that the structure difference for small pore size (d* ≤ 0.5) is too small to be seen by electrons in their moving.

## Nomenclature

*a*, Distance between two adjacent pores; *d*, Side length of a square nanopore; EMFP, Electron mean free path; ETC, Electronic thermal conductivity; *k*_e_, ETC; *l*_0_, Bulk EMFP; *l*_e_, EMFP of MNM; LTC, Lattice thermal conductivity; MNM, Metallic nanoporous materials

### Greek Symbols

*φ*, Porosity

### Superscript

*, Dimensionless quantity scaled by that of the corresponding bulk
